# CMOS-compatible, AlScN-based integrated electro-optic phase shifter

**DOI:** 10.1515/nanoph-2024-0263

**Published:** 2024-07-24

**Authors:** Valerie Yoshioka, Jicheng Jin, Haiqi Zhou, Zichen Tang, Roy H. Olsson III, Bo Zhen

**Affiliations:** 6572 University of Pennsylvania, Philadelphia, PA 19104, USA

**Keywords:** electro-optic phase shifter, integrated photonics, photonic materials

## Abstract

Commercial production of integrated photonic devices is limited by scalability of desirable material platforms. We explore a relatively new photonic material, AlScN, for its use in electro-optic phase shifting and modulation. Its CMOS-compatibility could facilitate large-scale production of integrated photonic modulators, and it exhibits an enhanced second-order optical nonlinearity compared to intrinsic AlN, indicating the possibility for efficient modulation. Here, we measure the electro-optic effect in Al_0.80_Sc_0.20_N-based phase shifters. We utilized the TM0 mode, allowing use of the *r*_33_ electro-optic coefficient, and demonstrated *V*_*π*_ *L* around 750 V cm. Since the electro-optic response is smaller than expected, we discuss potential causes for the reduced response and future outlook for AlScN-based photonics.

## 1 Introduction

Integrated photonics promises control over light signals in small, chip-size packages, enabling signal processing with lower power consumption. An integral component in complex photonic devices is modulation, which allows dynamic control of light with a single chip. Information can be transferred between electrical and optical signals, acting as an interface between traditional electronic computers and low-loss optical fiber networks. While there are many methods to modulate light, ranging from thermo-optic to plasma dispersion, one of the most useful is electro-optic modulation based on the electro-optic (EO) or Pockels effect, as its fast intrinsic speed could allow terahertz bandwidth. Though experimental devices are still limited by factors like impedance mismatch and phase matching between RF and optical signals [1], they regularly achieve bandwidth around 10s of GHz [2], [3], [4], limited only by structural design. Well-designed modulators are capable of bandwidths exceeding 100 GHz [5], [6]. In addition to design, modulation efficiency relies on the strength of the electro-optic coefficient, which is material-dependent. However, material choice often requires a trade-off between production cost and device efficiency.

Materials with straightforward growth and fabrication processes can be produced on a larger scale, reducing cost. Typically this means using materials that can be grown with CMOS foundry techniques, which have already been well-developed and optimized. The classic example is silicon, as its CMOS-compatibility allows scalable fabrication of photonic devices. Its high refractive index contrast increases modal confinement and reduces footprint [7]. However, bulk silicon is centrosymmetric and thus cannot use the electro-optic effect for modulation. Silicon modulators are still possible via DC Kerr effect or plasma dispersion, but each have difficulties. For the DC Kerr effect, a large bias field is required to effectively enable electro-optic modulation, increasing power consumption [8]. Plasma dispersion modulators (PDMs) control free carrier density to adjust refractive index, resulting in two issues: (1) tuning index requires free carrier movement, introducing an intrinsic speed limit that greatly limits modulation bandwidth [9] and (2) carrier density also impacts absorption, introducing unwanted intensity modulation and making higher-order modulation schemes more difficult [10]. Approaches to improve speed often reduce modulation efficiency [7]. Removing chirp requires digital post-processing [11] or additional tuning with thermo-optic phase shifters, using more energy [9].

In comparison, materials with intrinsic electro-optic coefficients enable modulation that is intrinsically fast and chirp-free, with efficiency only limited by material properties and design. Lithium niobate (LN) is a reliable optical material with low loss, large electro-optic coefficient (*r*_33_ ∼ 31 pm/V [12]), and mature fabrication process. Its strong performance in electro-optic modulators (EOMs) has been well-documented [3], including operation at reasonably low voltages [13]. The main drawback in using LN is its dependence on wafer bonding to integrate the material with existing platforms like silicon-on-insulator (SOI) [3], [14], [15]. Since it cannot be grown directly using CMOS-compatible techniques, large-scale production is more difficult and expensive. Other materials like barium titanate (BaTiO_3_, BTO) boast even larger electro-optic coefficients (*r*_42_ = 923 pm/V [16]) though they often exhibit higher losses than LN [16]. Though integrating BTO on silicon platforms can be easier than LN processes, epitaxial growth usually requires a template layer of strontium titanate (SrTiO_3_, STO) and often occurs at temperatures exceeding 600 °C [17]. Polycrystalline BTO can be grown at lower temperatures but suffers a reduction in the effective electro-optic response [18], [19]. Wafer bonding can interface epitaxially grown BTO onto silicon platforms to avoid damage from high growth temperatures [20], but bonding is more difficult to implement in large-scale production. While these materials can enable highly efficient devices with great performance, their fabrication constraints can increase cost, especially in integrating photonic and electronic devices on larger scales.

Aluminum nitride (AlN) is one of the few CMOS-compatible materials that exhibits an intrinsic electro-optic effect. It can be sputtered directly at sufficiently low temperatures (<400 °C), does not contaminate CMOS equipment with undesirable elements, and has already been integrated with electronic devices in the context of microelectromechanical systems (MEMS) [15]. However, its electro-optic coefficient is lower than that of LN (*r*_13_, *r*_33_ ∼ 1 pm/V [21]), reducing device efficiency. A potential method to improve its electro-optic response is controlling material properties through the use of substitutional Sc atoms.

Introducing Sc in AlN softens the crystal lattice, enhancing piezoelectric coefficients [22] and second-order optical nonlinearity (*χ* ^(2)^) [23]. The higher the Sc concentration, the larger the enhancement, until around 43 % Sc. At this concentration, the crystal structure starts shifting from wurtzite to cubic structure, regaining centrosymmetry and eliminating these properties. Like AlN, AlScN can be grown at sufficiently low temperatures of 350 °C or less, enabling compatibility with back-end-of-line (BEOL) processing in CMOS foundries [24]. While the electro-optic effect is often considered as a linear process since the shift in refractive index scales linearly with the applied electric field, it can be related to *χ* ^(2)^ as it involves frequency mixing between a low-frequency applied electric field and the optical electric field [25]. Thus, enhancements in *χ* ^(2)^ from frequency mixing between optical signals could indicate a larger electro-optic response. In this work, we demonstrate electro-optic phase shifting using Al_0.80_Sc_0.20_N. We utilized integrated Mach–Zehnder interferometer (MZI) devices to detect slight shifts in refractive index from applied voltage and to measure *V*_*π*_ *L*.

## 2 Theory

To determine how an applied electric field affects refractive index in AlScN, we utilize the formulation for the electro-optic effect in wurtzite crystals [25], which can be expressed by:

(1) $$\Delta {\left(\frac{1}{n^{2}}\right)}_{i}=\left[\begin{array}{ccc} 0 & 0 & r_{13}\\ 0 & 0 & r_{13}\\ 0 & 0 & r_{33}\\ 0 & r_{51} & 0\\ r_{51} & 0 & 0\\ 0 & 0 & 0 \end{array}\right]\left[\begin{array}{c} E_{x}\\ E_{y}\\ E_{z} \end{array}\right]$$

By solving for refractive index, we can determine how an externally applied electric field shifts index. Due to in-plane polycrystallinity in our AlScN samples, the contributions from in-plane electric field components, *E*_*x*_ and *E*_*y*_, are expected to cancel on average due to opposing domain directions. As such, *r*_51_ should not affect the overall response of our device, and only the *E*_*z*_ component should result in a measurable electro-optic effect. With this simplification, the new refractive indices can be expressed as:

(2) $$n_{o}^{\prime }=n_{o}-\frac{1}{2}n_{o}^{3}r_{13}E_{z}$$

(3) $$n_{e}^{\prime }=n_{e}-\frac{1}{2}n_{e}^{3}r_{33}E_{z}$$

Both ordinary and extraordinary indices are affected by an applied *E*_*z*_ field, with *r*_13_ controlling the change in *n*_*o*_ and *r*_33_ affecting *n*_*e*_.

In AlN, *r*_33_ and *r*_13_ are both around 1 pm/V [21]. To predict how EO coefficients in AlScN are enhanced, we can relate optical nonlinearity $\chi_{\mathrm{ijk}}^{\left(2\right)}=2d_{\mathrm{ijk}}$ to electro-optic coefficients [26]. Using full notation, we can relate the two values as:

(4) $$r_{\mathrm{ijk}}=\frac{-4d_{\mathrm{ijk}}}{n_{i}^{2}n_{j}^{2}}$$

where *n*_*i*_ is the refractive index along the *i*-axis. For AlN, *d*_33_ is 5.1 pm/V [23], corresponding to *r*_33_ = 1.2 pm/V, which is similar to experimental values. For Al_0.80_Sc_0.20_N, we measured *d*_33_ through second harmonic generation in the telecom regime to be 42.5 pm/V [23]. Assuming *d*_33_ remains the same for the electro-optic effect as well, the corresponding *r*_33_ would reach 8.1 pm/V, which is about a factor of 8 larger than intrinsic AlN. However, as we show later, this assumption may not be valid.

## 3 Design

**Figure 1:**
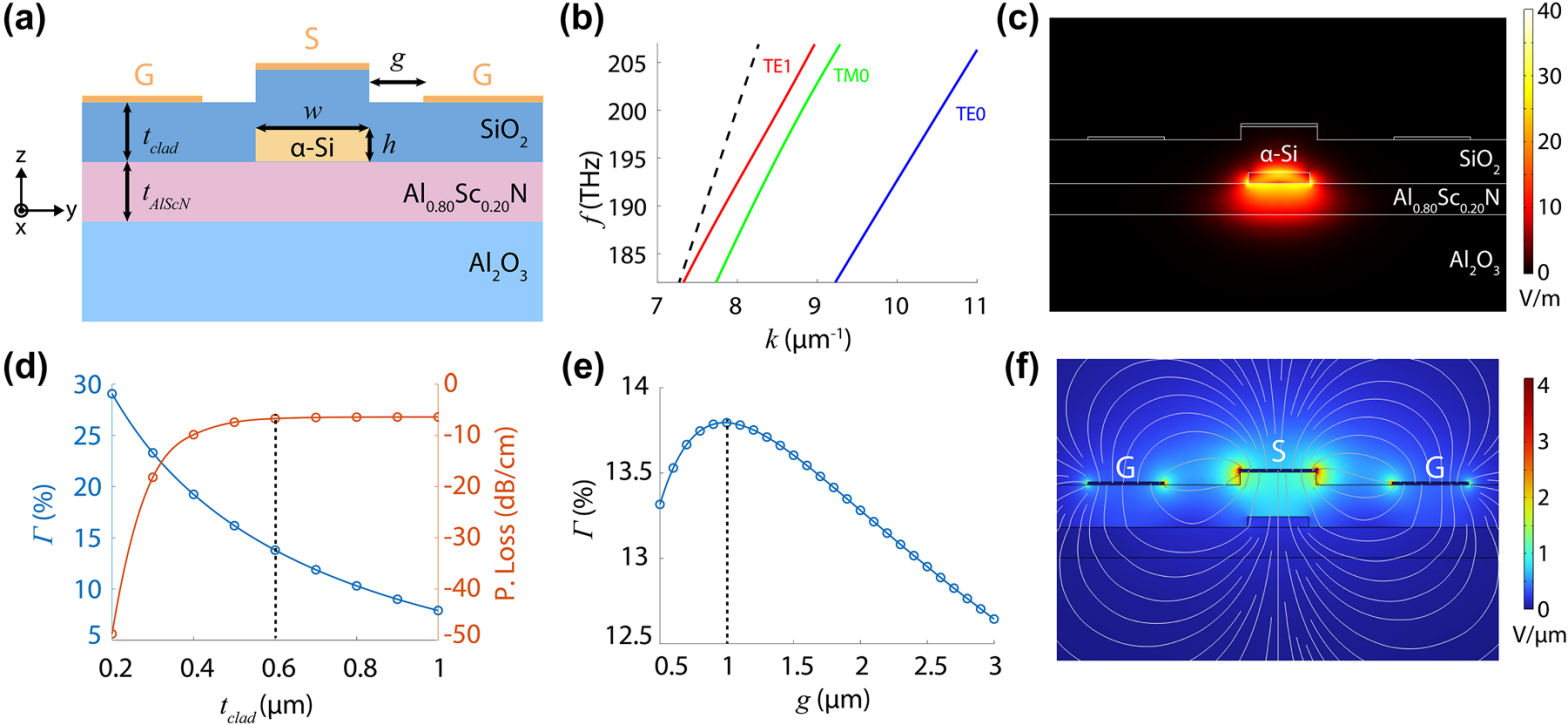
AlScN-based waveguide simulations. (a) Cross-section of waveguide structure with labeled materials and dimensions. Electrodes are comprised of gold and labeled with G for ground and S for signal. (b) Modal dispersion for TE0, TM0, and TE1 modes for *w* = 800 nm and *h* = 150 nm; all modes are below the light line (dotted black line) and therefore guided modes. (c) |*E*| in V/m for TM0 mode for *w* = 800 nm and *h* = 150 nm. A significant portion of the modal field is present in the AlScN layer. (d) Modal overlap Γ and waveguide propagation loss as a function of oxide thickness *t*_clad_; the final design used *t*_clad_ = 600 nm. (e) Modal overlap Γ as a function of electrode gap *g*; the final design used *g* = 1 μm. (f) Electric field in V/μm generated by applying voltage to the middle electrode in the GSG configuration; note that the field lines are primarily out-of-plane where the optical mode is located.

Our platform is comprised of a sapphire (Al_2_O_3_) substrate with $\left\langle 0001\right\rangle$ orientation, co-sputtered AlScN, an etched amorphous silicon (*α*-Si) waveguide, PECVD SiO_2_ cladding, and gold electrodes (Figure 1(a)). Measured AlScN thickness was 429 nm. We designed the devices to use the fundamental TM0 mode, which has an electric field directed along the extraordinary axis. As such, it enables use of the *r*_33_ electro-optic coefficient in AlScN, which is expected to be the larger coefficient. The *α*-Si waveguide has *w* = 800 nm and *h* = 150 nm. We chose these dimensions to balance good confinement in the AlScN layer with reasonable loss in fabricated devices. Based on the dispersion, we confirmed that the TM0 mode is guided (Figure 1(b)). Its electric field is also strongly localized in the AlScN layer despite the *α*-Si waveguide being used as the index contrast for guiding light (Figure 1(c)). While there is slight hybridization between the TM0 mode and TE1 mode at a waveguide width of 800 nm, we found that using a slightly wider waveguide reduced loss in fabricated devices. Since the mode is weakly confined in the etched silicon waveguide, a large bend radius around 250 μm was necessary to reduce bend loss.

We used coplanar electrodes in a ground-signal-ground (GSG) configuration to generate an out-of-plane electric field [21]. In order to generate a strong electro-optic response, the modal overlap between the applied electric field and optical mode needs to be large to encourage interaction between the fields. We define modal overlap as:

(5) $$\Gamma =\frac{g}{V_{in}}*\frac{\iint \left(E_{z,op}^{2}E_{z}\right)\mathrm{dyd}z}{\iint \left(E_{z,op}^{2}\right)\mathrm{dyd}z}$$

where *E*_*z*,*op*_ is the out-of-plane electric field of the TM0 optical mode, *E*_*z*_ is the applied out-of-plane electric field, *V*_in_ is voltage applied to the signal electrode, and *g* is the horizontal gap between electrodes. The integrals are evaluated in the region of the electro-optic material. Using overlap, we can define the effective out-of-plane field $E_{z}^{\prime }=\frac{V_{in}}{g}\Gamma$ , which induces change in refractive indices according to Eqs. (2) and (3). In order to maximize modal overlap, we optimized oxide thickness and the horizontal gap between the electrodes. Oxide thickness *t*_clad_ was set to be 600 nm to maximize effective electro-optic response, while keeping mode loss reasonable (Figure 1(d)). Electrode gap *g* was set to 1 μm to maximize modal overlap between the applied field and optical mode (Figure 1(e)). As a result, we can generate a strong out-of-plane field in the region of the waveguide, with reasonable modal overlap around 14 % (Figure 1(f)).

In order to detect a small index change induced by the electro-optic effect, we utilized Mach–Zehnder interferometers (MZIs). These devices work by splitting the input light into two branches, applying voltage to electrodes above one waveguide branch, and recombining the branches into a single output signal. Depending on wavelength, the light experiences either constructive or destructive interference, resulting in a pattern of fringes when wavelength is swept. The transmission, $T=\frac{I_{\text{out}}}{I_{in}}$ , at applied voltage *V*_in_ can be expressed as:

(6) $$T=\frac{T_{\max}}{2}\left[1+\cos\left(\frac{2\pi }{\lambda }\left(n_{\text{eff}}\Delta L-\Delta n_{\text{eff}}L_{1}\right)\right)\right]$$

where *L*_1,2_ are the path lengths for respective branches, with *L*_2_ = *L*_1_ + Δ*L*, *λ* is the guided wavelength, *T*_max_ is the maximum amplitude of the transmission based on total loss, *n*_eff_ is the effective index for the TM0 mode, and Δ*n*_eff_ is the index change induced in the *L*_1_ branch by voltage *V*_in_. Using the MZI transmission equation, we can solve for Δ*n*_eff_ by measuring the change in transmission, Δ*T* = *T*(0) − *T*(*V*_in_). Using the small angle approximation and the wavelength *λ*_0_ at which Δ*T* is maximized, we can express |Δ*n*_eff_| as:

(7) $$|\Delta n_{\text{eff}}|=\frac{\lambda_{0}\Delta T}{\pi L_{1}T_{\max}}$$

|Δ*n*_eff_| can then be related to *V*_*π*_ *L* by:

(8) $$V_{\pi }L=\frac{V_{in}\lambda_{0}}{2|\Delta n_{\text{eff}}|}$$

**Figure 2:**
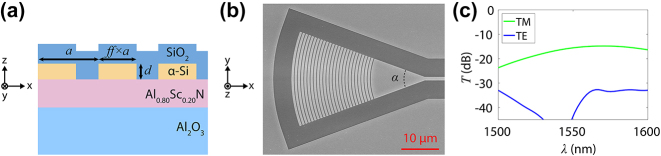
Grating coupler design and simulation. (a) Cross-section of grating design with period *a* = 0.87 μm. Fill factor *ff* = 60 % determines the relative width of the grating; with period *a* = 0.87 μm, the grating width is 0.52 μm. Etch depth *d* is 150 nm. (b) SEM image of top view of a fabricated grating coupler. Focusing angle *α* is 40°. (c) Simulated transmission for a single grating coupler. Maximum insertion loss for the TM0 mode is around −15 dB, while insertion loss for the TE0 mode is below −30 dB.

In order to couple light into and out of the integrated photonic device using a fiber array with an 8° polish angle, we designed grating couplers with focusing geometry [27]. We used grating period *a* = 0.87 μm and fill factor *ff* = 60 %, resulting in grating widths of 0.52 μm; we designed fully etched gratings with etch depth *d* = 150 nm to simplify the fabrication process (Figure 2(a)). A focusing angle of 40° was used (Figure 2(b)). By using grating couplers, we ensure mode selectivity as the grating period is tuned to only couple in light with the desired effective mode index (Figure 2(c)). 3D simulations indicate that insertion loss for a single grating coupler is about −15 dB for the TM0 mode. For the TE0 mode, insertion loss exceeds −30 dB across the wavelength range. As such, this grating coupler design ensures mode selective coupling.

## 4 Fabrication

Al_0.80_Sc_0.20_N was deposited onto the sapphire substrate via co-sputtering in a pure nitrogen environment using a pulsed DC physical vapor deposition system (Evatec CLUSTERLINE^®^ 200 II). Relative power applied to the Al and Sc targets was adjusted to control relative Sc concentration in the films. A thin seed layer was grown using 250 W on the Sc target and 875 W on the Al target to facilitate lattice matching to the substrate and ensure well-ordered crystal structure. Sc concentration was then linearly graded to achieve 20 % Sc concentration in the bulk layer. While polycrystalline in-plane, the c-axis is well-oriented perpendicular to the substrate. The full-width-half-maximum of the XRD curve is 1.476°, indicating excellent sample quality in the sputtered film. Its ordinary refractive index along the in-plane directions was measured to be 2.124 via prism coupling at 1550 nm, which is consistent with other refractive index measurements [28], [29]. Extraordinary index is oriented along the *c*-axis and is expected to be slightly larger, around 2.14 [28]. Loss at 1550 nm was measured via prism coupling to be 8.67 dB/cm, which is consistent with our prior measurements of similar samples [23]. AFM measurements indicate reasonable roughness, with *R*_*q*_ = 4.19 nm and *R*_*a*_ = 3.48 nm over a large area of 5 × 5 μm^2^.

**Figure 3:**
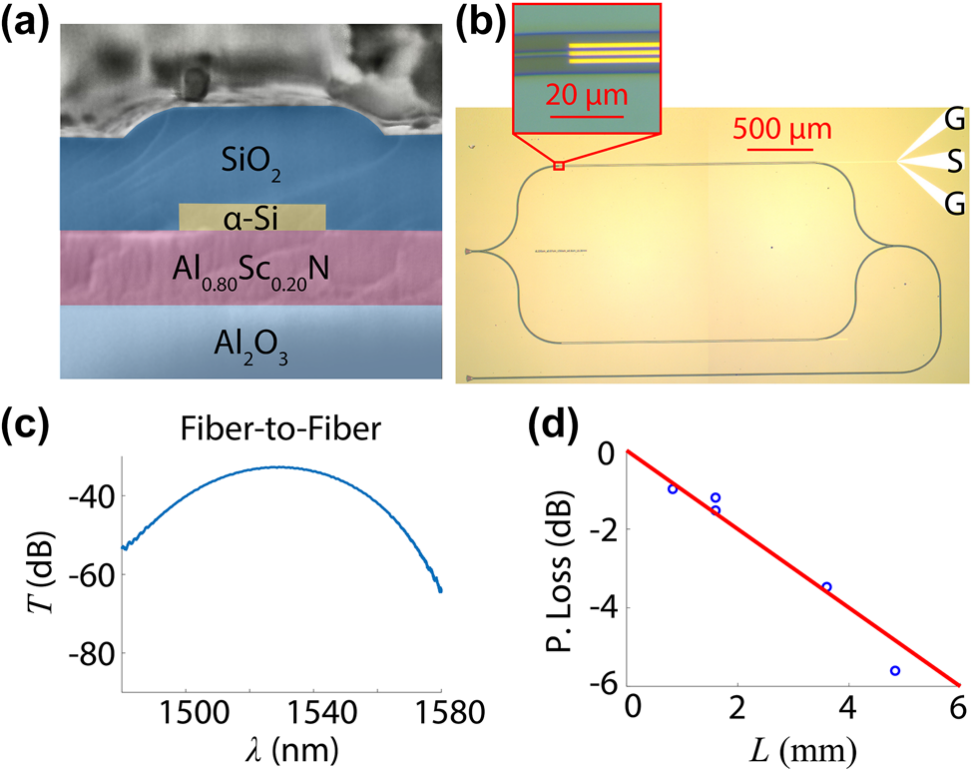
Fabricated AlScN-based photonic devices. (a) Cross-sectional SEM image of waveguide confirms expected dimensions. Colored shading is added to clarify materials in the structure; Al_2_O_3_is light blue, AlScN is pink, *α*-Si is yellow, and SiO_2_is dark blue. (b) Microscope image of an AlScN-based electro-optic phase shifter with *L*_1_ of 1.5 mm. Inset shows electrodes on top of the waveguide, visually confirming good alignment. Voltage is applied to the three electrodes in a ground-signal-ground (GSG) configuration. (c) Fiber-to-fiber transmission for a test device comprised of two grating couplers connected by a short waveguide. Insertion loss from a single grating coupler is around −15 dB, which is consistent with simulations. (d) Propagation loss for the waveguides is around 10 ± 2 dB/cm.

To make devices, intrinsic amorphous silicon (*α*-Si) was deposited on top of the AlScN layer via RF sputtering (Denton Explorer14 Magnetron Sputterer). Device patterns were defined using e-beam lithography (EBL). The e-beam resist (ZEP520A-07) was chemically developed using O-Xylene in a cold bath around −5 °C to −10 °C in order to reduce sidewall roughness. The *α*-Si was subsequently etched using CF_4_ in a dry reactive ion etching process (Oxford 80 Plus). After stripping the remaining resist using NMP in a heated ultrasonic bath, oxide was grown via PECVD as the top cladding layer, and the passive photonic response was measured. The thickness of each layer was confirmed by cross-sectional SEM (Figure 3(a)).

Electrode patterns were defined via EBL using PMMA resist. Square markers from the first EBL exposure enabled sufficient alignment between electrode and waveguide patterns. Once the electrode pattern was developed, we deposited Ti/Au via e-beam evaporation; a thin 5 nm layer of Ti was used to adhere the 40 nm layer of gold to the surface oxide in order to form the electrodes. Heated NMP was used to remove the remaining metal and resist, leaving the electrodes on top. Microscope images of a fabricated phase shifter device confirm good alignment between the deposited electrodes and underlying waveguides (Figure 3(b)).

To determine insertion loss, we fabricated test devices comprised of two grating couplers connected by a short waveguide. The total insertion loss of a test device was around −31 dB (Figure 3(c)), which corresponds to insertion loss around −15 dB per coupler and is consistent with simulated transmission. Though insertion loss could be improved through additional techniques such as apodization and shallow etching, this straightforward design is easier to fabricate and achieves sufficiently low loss in order to perform electro-optic measurements.

For propagation loss, we measured devices with different waveguide lengths and found loss was around 10 ± 2 dB/cm (Figure 3(d)). A few different factors contribute to this loss. In our devices, sidewall roughness is relatively low as cold development techniques and optimized etching recipes result in smooth features, reducing scattering loss. Surface roughness likely contributes to scattering loss but can be reduced through polishing. However, intrinsic material loss is fairly high, as Al_0.80_Sc_0.20_N exhibits an absorption loss of almost 9 dB/cm at 1550 nm based on prism coupling measurements. Strong modal confinement in the Al_0.80_Sc_0.20_N layer will therefore increase the propagation loss. While our waveguide loss is still reasonable, band gap is reduced with higher Sc concentrations [29], making absorption loss highly dependent on Sc concentration. As such, careful consideration of design and Sc concentration is necessary to achieve reasonable losses.

## 5 Results

To measure our devices, we used a telecom laser (Keysight 8164B) as the light source, connected to a 3-paddle fiber polarization controller. The fiber was then connected to an input on a fiber array in order to couple light onto the chip via grating couplers. The output light signal was directed to a power meter (Keysight N7744A) to determine transmission as a function of wavelength. We adjusted the chip position, chip rotation, and input polarization to maximize transmission for test devices of two grating couplers connected by a short waveguide. Once the positioning was optimized, we proceeded to measure more complex devices on the chip.

**Figure 4:**
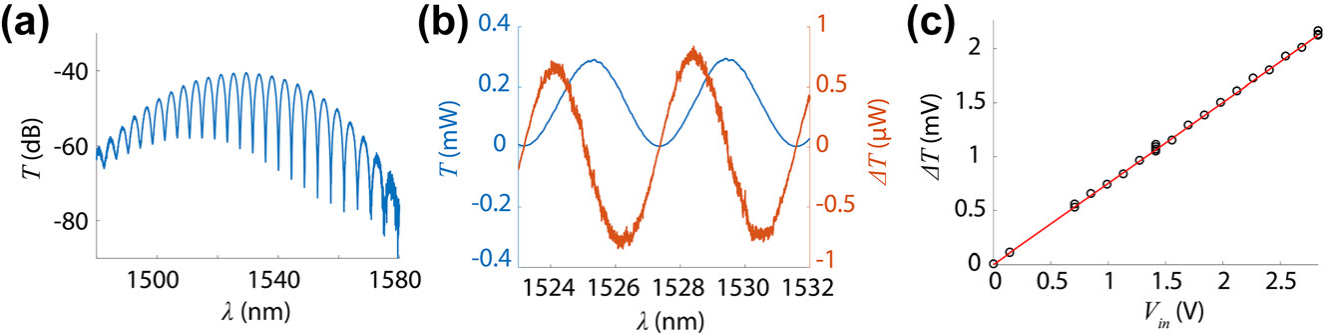
Electro-optic response of an AlScN-based phase shifter. (a) Transmission for a Mach–Zehnder interferometer with Δ*L* = 250 μm without applied voltage; free spectral range is around 4.2 nm. (b) Transmission *T* and change in transmission Δ*T* for an AlScN-based electro-optic phase shifter with effective length *L*_1_ = 1.5 mm on a linear scale for *V*_in_ = 10 V. Δ*T* reaches a maximum when *T* experiences its largest slope. (c) Linear increase in Δ*T* versus applied voltage *V*_in_, as measured by lock-in amplifier at 200 Hz.

The AlScN-based phase shifter was comprised of a Mach–Zehnder interferometer with an effective length *L*_1_ of 1.5 mm (Figure 3(b)). By applying voltage to the electrodes, an electric field is applied to the optical mode over this length on the upper branch of the MZI. We utilized a path length difference Δ*L* of 250 μm for our devices, resulting in a free spectral range around 4.2 nm (Figure 4(a)). The extinction ratio in the fringes varies between −20 and −30 dB, which is large enough to allow electro-optic measurements.

To determine our device’s performance, we applied DC voltage to the electrodes using an RF probe, functional from DC to 40 GHz, in direct contact with the chip. The probe was connected to a DC power supply to apply voltage. We measured the optical responses *T*(*V*_in_ = 0*V*) and *T*(*V*_in_ = 10*V*), observing a shift between the two due to the electro-optic effect (Figure 4(b)). The induced shift in transmission, Δ*T*, is maximized at the largest slope of the transmission signal, as expected. Δ*T* is about 400 times smaller than the maximum transmission, indicating a small electro-optic response. We confirmed that this response was due to the electro-optic effect by sweeping input voltage and measuring a linear trend in the response (Figure 4(c)). Due to the small signal, we used an InGaAs photodetector (Thorlabs PDA10DT) connected to a lock-in amplifier (Stanford Research Systems SR865A) to confirm this linear trend. Due to differences between the photodetector and power meter, Δ*T* was measured in volts using the lock-in amplifier and watts for DC measurements.

Based on DC measurements, we found our device experienced Δ*n*_eff_ = 8.2 × 10^−8^ per volt, which corresponds to a 3.4 pm wavelength shift after applying 10 V and *V*_*π*_ *L* = 933 V cm. Using more precise lock-in measurements with a driving frequency of 200 Hz, we found a slightly larger response of Δ*n*_eff_ = 1 × 10^−7^ per volt, corresponding to *V*_*π*_ *L* = 750 V cm. These responses are within 20 % of each other, with a smaller DC response. We also measured a second device at 100 Hz and found a similar response of Δ*n*_eff_ = 9.6 × 10^−8^ per volt, corresponding to *V*_*π*_ *L* = 800 V cm. In lock-in amplifier measurements, we primarily measured the EO effect around 100–200 Hz but found little variation in EO response between 1 Hz and 10 kHz.

The difference between the DC and lock-in measurements could be related to EO relaxation, which has been observed in low-frequency measurements of LN-based EOMs [3] and can be broadly attributed to free-carrier charges moving in the material [30]. For LN, where the effect is strongly present, conductivity is around 1 × 10^−6^ S/m. While AlN has very low conductivity around 1.1 × 10^−12^ S/m [31], the introduction of Sc in AlScN can increase conductivity to around 1 × 10^−7^ to 1 × 10^−5^ S/m [32]. There are methods to reduce the relaxation effect such as removing top cladding [33] or performing measurements at sufficiently high frequencies [3], but material interfaces, defects, and fabrication parameters can greatly complicate predictions of this effect [30]. However, it’s unclear how much the Sc concentration affects EO relaxation on different timescales.

## 6 Discussion

Based on our simulations, we expected Δ*n*_eff_ to be 2.8 × 10^−6^/V for Al_0.80_Sc_0.20_N based on its enhanced *χ* ^(2)^ or Δ*n*_eff_ = 4.4 × 10^−7^/V assuming *r*_33_, *r*_13_ = 1 pm/V. An MZI-based electro-optic phase shifter with AlN reported Δ*n*_eff_ = 2.4 × 10^−7^/V for the TM mode [34], which is similar to our expected performance for intrinsic AlN. However, our fabricated devices have low EO responses around Δ*n*_eff_ = 1 × 10^−7^/V, which are more similar to AlN and do not demonstrate any enhancement. There are a few possible explanations.

Some of the reduced performance comparing simulations to measurements is due to imperfections from fabrication. The overlap between electric field and optical mode could be smaller than expected due to slight variation in waveguide parameters or material properties. Invisible factors that cause uneven application of field energy, such as variation in the silicon or oxide thickness, roughness on the oxide surface, and imperfections in the electrodes, can similarly reduce EO response. If the waveguide height and width vary by 10 nm, Δ*n*_eff_ can be reduced by 5–10 %. Electrode alignment also plays a factor in total measured signal. Visually, the electrode is aligned within 1 μm of the waveguide, but even slight misalignments can reduce EO response. A lateral shift of 500 nm would reduce Δ*n*_eff_ by about 10 %. Note that the DC permittivity of AlScN also changes with concentration. For intrinsic AlN, *ϵ*_*r*_ is around 9.9, while for Al_0.80_Sc_0.20_N, *ϵ*_*r*_ increases to 13.7 [28]. Higher permittivity reduces electric field in the material, reducing overall efficiency by about 25 % compared to intrinsic AlN, assuming electro-optic coefficients are comparable. However, these factors have relatively small effects even when combined.

In terms of sample quality, a film of surface oxidation on the AlScN layer could reduce overlap and thus overall response, particularly since the optical mode is concentrated at the top surface of the AlScN. There is also some evidence that oxidation can extend into the bulk of the sputtered AlScN when the crystal structure exhibits many grain boundaries [35]. Since oxidation depends on film structure, its extent varies depending on growth method and sample quality. Based on prior studies [35], the surface oxidation in our samples is likely around 10–20 nm. As a conservative estimate, a 20 nm layer of surface oxidation with the same refractive index as AlScN but no electro-optic response would reduce Δ*n*_eff_ by about 13 %. However, it is unclear how surface oxidation in AlScN affects performance since other material properties such as refractive index or DC permittivity could be affected. Regardless, methods to prevent or remove this oxidation would likely improve performance in future devices. Fully etched waveguide designs may also have better performance as they allow mode overlap to be localized further away from oxidized surface areas.

In terms of intrinsic limitations, a recent paper detailing the theoretical electro-optic coefficients in AlScN corroborates our observation of a small electro-optic response [36]. Based on their DFT calculations, the predicted electro-optic coefficients for intrinsic AlN are *r*_33_ = 1.7 pm/V and *r*_31_ = −1 pm/V; *r*_15_ is close to 0 pm/V. In comparison, the calculated coefficients for Al_0.80_Sc_0.20_N are around *r*_33_ = 1 pm/V, about half of the value predicted for intrinsic AlN, and *r*_31_ = 0.2 pm/V; *r*_15_ increases to around 1 pm/V, but we expect its contribution in our device is negligible. As such, the small response in our Al_0.80_Sc_0.20_N phase shifters can be attributed to a decrease in electro-optic coefficients at this Sc concentration.

There is potential for significant enhancement in electro-optic response at higher Sc concentrations, with predicted coefficients of *r*_33_ around 50 pm/V and *r*_31_ around 10 pm/V for Al_0.50_Sc_0.50_N [36]. However, fabricating films with the necessary uniform crystal structure is difficult at these concentrations, and waveguide loss is likely much higher due to decreased band gap. Furthermore, the predicted enhancement is attributed to piezoelectric contributions, which may be reduced by clamping the waveguide between substrate and cladding or increasing the driving frequency past acoustic resonances. The implementation of a more efficient electro-optic modulator based on AlScN is possible but faces significant challenges in design and fabrication.

## 7 Future outlook

While this work demonstrates the difficulty in using AlScN for electro-optic modulation, modern fabrication advances could improve response. Previously, AlScN etching recipes were primarily limited by sidewall roughness [37]. To avoid this loss, we used strip-loaded silicon waveguides to guide light, as silicon etching recipes are better established and more reliable. While these waveguides still allow some interaction with the AlScN film underneath, the modal confinement in AlScN is limited. However, improvements in etching recipes over the past few years have enabled directly etched AlScN waveguides with smoother sidewalls [38], [39]. As a result, light can be more strongly confined in AlScN to improve nonlinear response without concerns about sidewall roughness or surface oxidation.

Intrinsic material loss, due to higher absorption with Sc concentration and polycrystalline structure, remains a significant limitation. However, the concentration of Sc can be controlled depending on the tolerance for loss. As for crystalline quality, there are methods for epitaxial growth of single crystal AlScN films, which could reduce loss regardless of Sc concentration [40]. Techniques like polishing and annealing have also been demonstrated to reduce propagation loss [38]. Much lower waveguide losses have been recently reported, indicating waveguide losses below 2 dB/cm in etched Al_0.70_Sc_0.30_N rib waveguides [39]. While their data suggests lower intrinsic loss, potentially due to their growth technique, their waveguides are also quite thin, reducing confinement in lossy AlScN. By adjusting waveguide design, confinement in AlScN could be reduced in passive guiding areas of a device to decrease loss and then increased in nonlinear or electro-optic areas to improve efficiency. Additional studies on growth of low-loss AlScN films would greatly improve future performance for AlScN-based photonics.

Additional flexibility in growing AlScN on different substrates could also improve overall response. In our devices, we used sapphire as a substrate to ensure highly oriented AlScN films due to lattice matching considerations, but as a result, we were unable to fabricate devices with vertical electrodes and used a less efficient coplanar design instead. Recent studies have used a thin seed layer of AlN to grow AlScN on oxide [41], which is easier to work with and can be deposited on a variety of substrates. It would also allow the possibility of depositing a bottom electrode beneath the lower oxide layer for vertical electrodes, improving overlap between optical and electrical fields and thus overall response.

Finally, periodic poling in AlScN could allow quasi-phase-matching for frequency mixing applications. Demonstrations of poling in Al_0.68_Sc_0.32_N have achieved poling widths as narrow as 250 nm [42]. While poling in AlN has been demonstrated at higher temperatures [43], it is a relatively recent implementation and has not yet been widely utilized in integrated photonic devices. Thus, the introduction of Sc could facilitate room temperature poling to improve efficiency in CMOS-compatible, nonlinear integrated devices.

## 8 Conclusions

We designed, fabricated, and measured electro-optic phase shifters based on AlScN. Phase shifters with Al_0.80_Sc_0.20_N had a measured performance of *V*_*π*_ *L* = 750 V cm. We expect developments in AlScN fabrication techniques and phase shifter design have the potential to improve future AlScN-based modulators. While further studies are needed to shed light on the intrinsic limits of AlScN, from its loss to its electro-optic response, its CMOS-compatibility and enhanced optical nonlinearity could still facilitate large scale production of other nonlinear integrated photonic devices.
